# Follicular fluid proteomic alterations associated with oocyte developmental potential in polycystic ovary syndrome

**DOI:** 10.3389/fendo.2026.1784682

**Published:** 2026-04-16

**Authors:** Ling Hong, Chunyu Huang, Meilan Mo, Xuejin Wang, Qing Sun, Ruiteng Zhang, Su Liu

**Affiliations:** 1 Shenzhen Key Laboratory of Reproductive Immunology for Peri-implantation, Shenzhen Zhongshan Institute for Reproductive Medicine and Genetics, Shenzhen Zhongshan Obstetrics & Gynecology Hospital, Shenzhen, China; 2 Guangdong Engineering Technology Research Center of Reproductive Immunology for Peri-implantation, Shenzhen, China

**Keywords:** cholesterol metabolism, endoplasmic stress, follicular fluid, oocyte quality, polycystic ovary syndrome

## Abstract

**Introduction:**

PCOS is the most common disorder encompassing reproductive, metabolic, and endocrine abnormalities, and has an increased risk of adverse pregnancy and neonatal complications, such as miscarriage, gestational diabetes mellitus, and preterm birth. Many studies suggested that oocyte quality is compromised in patients with PCOS compared to those without PCOS. To systematically characterize the follicular fluid (FF) proteomic landscape and identify potential biomarkers for oocyte quality assessment, we conducted a label-free proteomics analysis between patients with and without PCOS.

**Methods:**

Gonadotropin-releasing hormone antagonist was used for ovarian stimulation. FF was collected by transvaginal ultrasound-guided aspiration 36 h after human chorionic gonadotropin administration, followed by centrifugation at 800 x g for 10 min at 4°C, and then the supernatants were collected for proteomic analysis. Embryo outcome was compared using a Cox model analysis adjusted for age, BMI and duration years of infertility. The fundamental procedure of proteomics included depletion of highly abundant proteins, extraction of proteins, filter aided proteome preparation, desalination of peptides, LC-MS/MS analysis, and bioinformatics analysis. ELISA assay was employed to validate the expression levels of proteins in FF.

**Results:**

Our study has demonstrated that the normal fertilization rate among PCOS patients was markedly lower compared to controls when adjusted for age, body mass index (BMI), and duration of infertility. The proteomics analysis has identified 11 upregulated and 17 downregulated DEPs in the PCOS group, notably, VNN1 expression was upregulated, while PLTP and HYOU1 were downregulated. The proteomic sequencing results categorized according to the quality rate of D3 embryos revealed that DEPs in FF were significantly enriched in cholesterol metabolism. Oocyte quality declines in association with abnormal PLTP and cholesterol metabolism, and activated endoplasmic stress in FF of PCOS.

**Discussion:**

PCOS patients exhibit significantly lower fertilization rate compared to the controls. Our findings delineate a distinct proteomic signature in PCOS FF featuring downregulated PLTP and HYOU1 concomitant with VNN1 overexpression, implicating their pivotal involvement in the pathophysiological mechanisms underlying oocyte competence impairment. Furthermore, the observed correlation between dysregulated lipid homeostasis and compromised oocyte developmental potential suggests a mechanistic link between follicular microenvironment alterations and reproductive outcomes in PCOS.

## 1 Introduction

PCOS is the most common disorder encompassing reproductive, metabolic, and endocrine abnormalities, with a prevalence between 5% and 10% (1), which is characterized by oligo-ovulation or anovulation, clinical and/or biochemical hyperandrogenism, and polycystic ovarian morphology as observed by ultrasound. PCOS also represents one recognized cause of female infertility, mainly due to chronic anovulation (2, 3). It was reported that patients with PCOS undergoing IVF have an increased risk of adverse pregnancy and neonatal complications, such as miscarriage, gestational diabetes mellitus, and preterm birth (4). Many studies suggested that oocyte quality is compromised in patients with PCOS compared to those without PCOS (5). Our previous study showed that while patients with PCOS undergoing IVF tend to have more oocytes collected per retrieval, they exhibit lower fertilization rates, although pregnancy and live birth rates are comparable to those in control patients (6). Alterations in oocyte competence may be the causative factor for infertility and increased adverse pregnancy outcomes. However, the specific changes in oocytes responsible for adverse outcomes, as well as the underlying mechanisms in PCOS, remain inadequately understood.

Oocyte quality is rate-limiting for female fertility, playing a crucial role during the fertilization process and subsequent embryo development (7). However, available tests estimate ovulation (e.g., luteal phase serum progesterone) and oocyte numbers (e.g., anti-Müllerian hormone (AMH), follicle stimulating hormone (FSH) and antral follicle counts (AFC)), but do not adequately evaluate the third, and most pivotal, oocyte-specific parameter: quality (8). To date, there are still no specific biomarkers of poor oocyte quality other than through IVF treatment, when readouts of oocyte quality such as preimplantation embryo development can be assessed (9). Recently, the accurate evaluation of oocyte quality through the chemical composition of FF is has gained significant attention in clinical assisted reproductive therapy. FF is a liquid composed of secretions produced, primarily containing hormones, enzymes, anticoagulants, electrolytes, reactive oxygen species and antioxidants, which fills the follicular antrum, facilitating cell communication within the antral follicle and providing nutrients to the oocyte (10). FF creates a microenvironment essential for the development of oocytes and plays an important role in the physiological processes of follicle growth, oocyte maturation, and ovulation (11). Certain biochemical characteristics of the follicular fluid surrounding oocytes may significantly influence oocyte quality, fertilization, and embryonic development, making it essential to investigate potential biomarkers within FF.

Proteomic analysis has been utilized to identify critical proteins and to deepen our comprehension of the physiological mechanisms underlying follicular development and oocyte maturation. Recent proteomic investigations employing mass spectrometry-based methodologies have revealed alterations in protein profiles within the FF of PCOS patients. These alterations encompass proteins linked to a variety of biological processes, such as the complement system, extracellular matrix dynamics (12), lipid metabolism (13), cholesterol metabolism (14) and hormonal regulation (15), providing evidence of pathological alterations in complement activation, angiogenesis (16), extracellular matrix regulation, lipid metabolism, immune function, and hormone regulation in the follicular microenvironment in PCOS (17). Nonetheless, these findings do not clarify why certain PCOS patients struggle to produce viable oocytes and high-quality embryos following ovulation induction. It appears that additional direct evidence is required to illustrate the pathological alterations in oocyte quality and the microenvironment of follicular development in this specific subgroup of PCOS patients. Further studies are required to verify the associations between alterations in follicular fluid components and variations in oocyte quality among individuals with PCOS.

To date, a comprehensive profiling of FF proteomes from oocytes capable of developing into high-quality embryos has not been thoroughly investigated. To the best of our knowledge, the proteomic profiles in the FF from PCOS patients with and without high-quality embryos undergoing IVF treatment have not been reported. In this study, the patients, divided into PCOS and control groups, were further subdivided into four subgroups: controls with a high rate of high-quality D3 embryos (HC), controls with a low rate of high-quality D3 embryos (LC), PCOS with a high rate of high-quality D3 embryos (HP), and PCOS with a low rate of high-quality D3 embryos (LP). Therefore, the present study was undertaken to determine whether the human FF proteome is altered between PCOS patients and controls, and between PCOS subgroups with varying rates of high-quality D3 embryos, following the same controlled ovarian stimulation (COH). For this purpose, we analyzed the global changes in proteins and biological pathways associated with PCOS, and the presence of D3 embryo quality that may reveal potential protein biomarkers of oocyte quality in PCOS.

## 2 Material and methods

### 2.1 Subjects

This study consisted of patients who underwent IVF/ICSI treatment at the Reproductive Center, Shenzhen Zhongshan Obstetrics & Gynecology Hospital. PCOS was diagnosed using the Rotterdam criteria (2003), which required at least two of the following conditions: clinical and/or biochemical evidence of hyperandrogenism, oligo- and/or anovulation, and polycystic ovarian morphology, and excluded other causes of hyperandrogenism and ovulation dysfunction. The control group consisted of infertile women undergoing IVF/ICSI treatment due to tubal or male factors that met the following inclusion criteria: normal ovarian reserve (regular menstrual cycles, FSH < 10 IU/L, and AMH ≥ 1.5 ng/ml), no signs of hyperandrogenism, no endocrine diseases, and normal ovarian and uterine morphology confirmed by ultrasound. All recruited women were ≤ 40 years old and had a BMI ranging from 18 to 30 kg/m^2^. Women with a history of ovarian surgery, uterine malformations, thyroid disease, and chromosomal abnormalities were excluded. Gonadotropin-releasing hormone antagonist was used for ovarian stimulation.

We identified 38 women with PCOS and 42 regularly menstruating control women. The clinical and biochemical characteristics of all participants are listed in **Table 1**. In order to mitigate variations attributable to the quantity of oocytes retrieved, we selected participants who had more than 10 oocytes retrieved and obtained their follicular fluid for the following proteomic sequencing analysis. Among these women, 20 were included for proteomic sequencing as screening: 10 non-PCOS controls, five controls with a high rate of high-quality D3 embryos (HC) and five controls with a low rate of high-quality D3 embryos (LC); 10 PCOS patients, five PCOS with a high rate of high-quality D3 embryos (HP) and five PCOS with a low rate of high-quality D3 embryos (LP). The high-quality D3 embryos are defined as those originating from normally fertilized oocytes, characterized by the presence of 7 to 9 embryonic cells on the third day post-fertilization, and exhibiting a fragmentation rate of less than 10%, which were determined by the Chinese Medical Association Reproductive Medicine Branch (**CSRM, 2019**). According to data obtained from the 2015 China CSRM data reporting system, the average rate of high-quality D3 embryos for IVF/ICSI cycle is 44.10% and 42.82%, respectively. We establish a threshold for high-quality D3 embryos at a rate exceeding 70%, while a rate below 30% is classified as indicative of low-quality D3 embryos.

**Table 1:** The baseline characteristics of patients with and without PCOS.

Characteristics	Control(n=42)	PCOS (n=38)	*P*
Maternal age	31.00±3.76	29.92±3.89	0.212
Maternal BMI (kg/m^2^)	21.44±3.05	21.85±3.03	0.545
Basal FSH level (IU/L)	6.01±2.46	6.64±2.16	0.237
Basal LH level (IU/L)	5.54±3.42	9.47±7.59	0.004
LH/FSH ratio	0.93±0.50	1.40±0.90	0.005
Basal E2 level (pg/mL)	60.70±10.39	58.97±8.59	0.898
Basal P level (ng/mL)	2.52±0.98	0.52±0.09	0.050
Basal PRL level (pg/mL)	42.70±12.05	45.30±15.17	0.893
Total T (ng/mL)	0.42±0.38	0.66±0.52	0.033
TSH (μIU/mL)	1.87±1.27	2.01±1.05	0.652
Fasting glucose(mmol/L)	5.75±0.61	5.54±0.60	0.153
Fasting insulin (μU/mL)	18.34±5.22	14.99±2.14	0.483
AMH (ng/mL)	4.54±2.14	8.08±4.16	0.000

BMI, body mass index; FSH, follicle stimulating hormone; LH, luteinizing hormone; E2, estradiol; P, progesterone; PRL, prolactin; T, testosterone; TSH, thyroid stimulating hormone; AMH, anti-Müllerian hormone.

### 2.2 Follicular fluid samples collection

FF samples from 10 patients with PCOS and 10 controls were collected for proteomic analysis. Gonadotropin-releasing hormone antagonist was used for ovarian stimulation. Follicular fluid was collected by transvaginal ultrasound-guided aspiration 36 h after human chorionic gonadotropin administration. Only clear fluid without blood or flushing medium contamination was collected. After oocyte isolation, FF samples were centrifuged at 800 x g for 10 min at 4°C to remove cellular components and debris. The supernatants were stored at -80°C before further processing. To further validate the identified proteins, additional FF samples from 42 patients with PCOS and 38 controls were analyzed by ELISA.

### 2.3 Laboratory tests

Baseline hormones were detected on days 2–5 of the menstrual cycle in all recruited subjects. For each participant, peripheral blood was collected in the morning after an overnight (8–10 hours) fast preferably on cycle day 3 of a natural menstrual cycle in regularly menstruating women or during withdrawal bleeding in amenorrheic women. For PCOS patients within oligo- or anovulation, hormone levels were measured when follicle size was < 1.0 cm and endometrial thickness < 0.7 cm by ultrasound. Basal serum levels of luteinizing hormone (LH), FSH, thyroid stimulating hormone (TSH), total testosterone (T) and AMH were measured on menstrual cycle day 2 or 3 in control and PCOS patients by chemiluminescence under Cobas e601 (Roche Diagnostics, Germany) using commercial kits, whereas plasma glucose and other biochemical parameters were assayed on Cobas c501 autoanalyzer (Roche Diagnostics, Germany). For all measurements, the inter- and intra-assay coefficient of variations were within the limits permitted by the manufacturers.

### 2.4 Sample preparation and depletion of highly abundant proteins

All samples were centrifuged at 10,000 rpm for 10 min at 4 °C, then 10 μl of supernatant sample was depleted with High Select™ Top14 Abundant Protein Depletion mini column (Thermo Fisher Scientific, USA, A36371), and incubation was carried out for 20 min at room temperature. The supernatant was collected by centrifugation at 1,000g for 2 min, and acetone was added to a 6:1 ratio (acetone to supernatant). Incubated the mixture at -20°C overnight, then centrifuged to collect the protein pellet. Washed the pellet and redissolved it in buffer (300 mM triethylammonium bicarbonate, TEAB, 6M guanidine hydrochloride), following by centrifuging 13000 rpm for 10 min at 4°C. Protein concentration was determined by BCA assay.

Equilibrate the high abundance protein chromatography column (ThermoFisher Cat. A36370) to room temperature for at least 30 minutes in advance. After thawing the sample on ice, centrifuge the samples at 4 °C and 10000 rpm for 10 minutes, then extract 10 μL of the middle layer sample. Remove the chromatographic column nut and directly add samples to the resin slurry in the chromatographic column. Cover the chromatography column and invert it several times until the resin is uniformly distributed in the solution. At room temperature, gently rotate and invert the mixer and incubate for 20 minutes. Ensure that the sample is mixed with the resin during incubation. Alternatively, gently swirl the column every few minutes. After incubation, break the bottom cover (gallowing the liquid to flow out). Loosen the top cover above the centrifuge tube to connect the top and bottom. Place the micro column into a 2ml collection tube, centrifuge at 1000 × g for 2 minutes, and remove the chromatography column containing the resin. The filtrate, now depleted of albumin, IgG, and other high abundance proteins, is ready for further processing or storage at -20 °C for future use.

### 2.5 Extraction of proteins

Six times the volume of 100% acetone was added into the pretreated samples and then precipitate it overnight at 20 °C. The next day, the precipitate was obtained by centrifugation at 13000 rpm for 15 minutes at 4 °C. The precipitate was then washed twice with 500 μL of a pre-cooled solution (ethanol: acetone: acetic acid=50:50:0.1). After washing, the precipitate was dissolved in 6 M guanidine hydrochloride and 300 mM TEAB. The sample concentration was measured, and the solution was stored in a refrigerator at 4 °C. A portion of the sample was diluted to determine the concentration using the BCA method.

### 2.6 Filter aided proteome preparation

According to the measured protein concentration, take the same quality protein from each sample, and dilute different groups of samples to the same concentration and volume with 25 mM NH_4_HCO_3_. Then, 1 M Dithiothreitol was added (terminal concentration 20 mM), and the reduction reaction was kept for 1 h at 57 °C. Subsequently iodoacetamide was added (terminal concentration 90 mM) and incubated for 40 min at room temperature under dark conditions. The sample solution was centrifuged on a 10 KDa ultrafiltration tube at 12000 rpm, and ammonium bicarbonat was added into the ultrafiltration tube to wash 4 times. According to the amount of protein, add the corresponding volume of enzymolysis diluent (protein: enzyme = 30:1 (m/m)), to redissolve the protein precipitate, then the solutions were incubated for digestion at 37 °C for 12 h. Next day the peptides were collected after centrifugation, and dried by centrigugal concentration.

### 2.7 Desalination of peptides

After centrifuging and concentrating the dried peptides, desalinate them on a Monospin column, and prepare them for mass spectrometry analysis. Dissolve the dried mixed peptide segments in a 0.1% trifluoroacetic acid (TFA) solution. Activate the desalination column with 100% acetonitrile. Equilibrate the desalination column with a 0.1% TFA solution. Add the redissolved sample to the desalination column and centrifuge it; wash the desalination column with a 0.1% TFA solution. Elute the peptide segments with a 50% acetonitrile solution, centrifuge, wash off the peptide segment, and collect the eluate using a new EP tube. Centrifuge the eluate to concentrate it, and dry it to remove the acetonitrile.

### 2.8 LC-MS/MS analysis

The qualitative and quantitative analysis of proteins were performed on an Orbitrap Fusion Lumos mass spectrometer (Thermo Scientific, USA) coupled with EASY-nLC 1000 liquid chromatography system (Thermo Scientific, USA). The vacuum-dried samples were reconstituted with 0.1% formic acid (FA), and separated on a 75 μm I.D. × 25cm C18 home-made analytical column (C18, 1.9 μm, 120 Å, Maisch GmbH, Germany). The rate of flow was 300 nL/min. Data was acquired with full scans (m/z 350-1800) at a mass resolution of 60,000 (FWHM). The precursor ions were fragmented in the high energy collision dissociation at normalized collision energy of 30%.

### 2.9 Bioinformatics analysis

The raw files obtained from Orbitrap Fusion Lumos mass spectrometer were analyzed by Proteome Discoverer 2.4 (Thermo Scientific, USA), and the spectra were searched against the human UniProt protein database. Fixed modifications: Carbamidomethyl(C), Variable modifications: Oxidation(M) and Acetyl(N-Term); Trypsin digestion was selected to allow two missing sites. The false positive rate of peptide segment (FDR) was less than 5%. For Gene Ontology (GO) and KEGG pathway enrichment analyses, the Benjamini–Hochberg false discovery rate (FDR) method was applied to correct raw p-values for multiple testing across all functional categories. Enriched terms or pathways with an adjusted p-value < 0.05 were considered statistically significant.

### 2.10 Weighted gene co-expression network analysis

Weighted protein co-expression network analysis was specifically performed on follicular fluid using the R package WGCNA (V1.68) (18). The expression matrix was restricted to only expressed proteins, when the number of reads sequenced was greater than 10 in a minimum of 20 samples, normalized for samples depth (count per million reads, CPM), and log-transformed, including a pseudo-count of 4 (log_10_(CPM + 4)). Then, the optimal soft threshold for adjacency computation was graphically determined, and we plotted module detection via dynamic tree cutting. For the demonstration of the relationship between different modules, results were visualized using module and eigengene relation heatmap, and the protein co-expression network was extracted and further processed using MCODE in Cytoscape software (V3.6.3) (19). For a distinct and precise representation of the data, proteins contained in the significantly relevant modules were extracted and analyzed using Metascape (20).

### 2.11 ELISA validation

In addition to the 20 sequenced samples, extra 60 (including 28 PCOS and 32 controls) were added to measure relative follicular fluid protein expression using ELISA for validation. The concentrations of selected proteins in follicular fluid samples were measured by commercial ELISA (Enzyme-Linked ImmunoSorbent Assay, ELISA) kit: PLTP (ab289907, Abcam), IGFBP1 (ab233618, Abcam), M-CSF1 (ab245714, Abcam), NCAM1 (CSB-EL015511HU, Cusabio), HYOU1 (EH3246, FineTest), and VNN1 (CSB-EL025883HU, Cusabio) referring to the manufacture’s protocol.

### 2.12 Statistical analysis

Statistical analysis was performed using SPSS software (IBM Corp., Armonk, NY, United States) and GraphPad Prism 8 software (GraphPad Software, SanDiego, California). Continuous variables were expressed as the mean ± standard deviation. Differences between groups were compared using the two-tailed Student’s t-test. Variables with skewed distribution were presented as the median (inter-quartile range) and compared using a nonparametric test. Correlations between different variables were determined using Pearson correlation analysis. Embryo outcome was then compared between PCOS and matched control patients using a Cox model analysis adjusted for additional baseline covariates (age, BMI and duration years of infertility).

## 3 Results

### 3.1 Decreased fertilization rate in PCOS patients

To evaluate oocyte and embryo development outcomes in patients with PCOS, we conducted a comparative analysis between PCOS and control groups. We found that there was a significant increase in both the number of antral follicles and the metaphase II (MII) oocytes rate among PCOS patients when compared to the control group, after controlling for variables such as age, BMI, and duration of infertility through propensity score matching. However, the normal fertilization rate was markedly lower in the PCOS group (83.68% vs. 76.41%, *P* < 0.001) (**Table 2**).

**Table 2:** The characteristics and embryo outcomes of patients with and without PCOS.

Characteristics	Control (n=355)	PCOS (n=355)	*P*
**Maternal age**	30.57±4.42	30.74±3.86	0.587
**Infertility duration (y)**	3.45±2.65	3.59±2.56	0.464
**BMI (kg/m^2^)**	22.18±3.41	22.40±3.31	0.378
**COH**			<0.001
GnRH-a	76.90%	47.04%	
GnRH-A	20.28%	52.11%	
Other	2.82%	0.85%	
**Dose of gonadotropin (IU)**	2.10±0.76	2.11±0.70	0.789
**Duration of gonadotropin stimulation (d)**	9.84±3.63	9.90±2.27	0.795
**Total dose of gonadotropin (IU)**	25.16±10.61	24.05±9.48	0.143
**Serum E2 level (pg/ml) on hCG day**	2665.79±1317.90	3089.26±1572.76	<0.001
**Serum P level (pg/ml) on hCG day**	0.60±0.29	0.59±0.28	0.950
**Serum LH level (pg/ml) on hCG day**	2.53±3.52	3.41±3.54	<0.001
**Numbers of retrieved oocytes**	14.53±6.00	19.08±8.80	<0.001
**Cycles with different technologies**			<0.001
IVF	27.04%	88.17%	
ICSI	72.96%	11.83%	
**Rate of MII oocytes (%)**	87.14%	91.54%	<0.001
**Rate of fertilization (%)**	87.40%	88.68%	0.185
**Rate of cleavage embryos (%)**	97.18%	97.14%	0.928
**Rate of D3 high-quality embryos (%)**	58.49%	57.28%	0.505
**Rate of normal fertilization (%)**	83.68%	76.41%	<0.001
**Rate of embryo formation (%)**	48.66%	46.83%	0.396
**Rate of available blastocyst (%)**	42.79%	40.72%	0.320
**Rate of high-quality blastocyst (%)**	24.41%	21.46%	0.082

### 3.2 Identification and functional analysis of differentially expressed proteins in follicular fluid of PCOS patients

**Figure 1:**
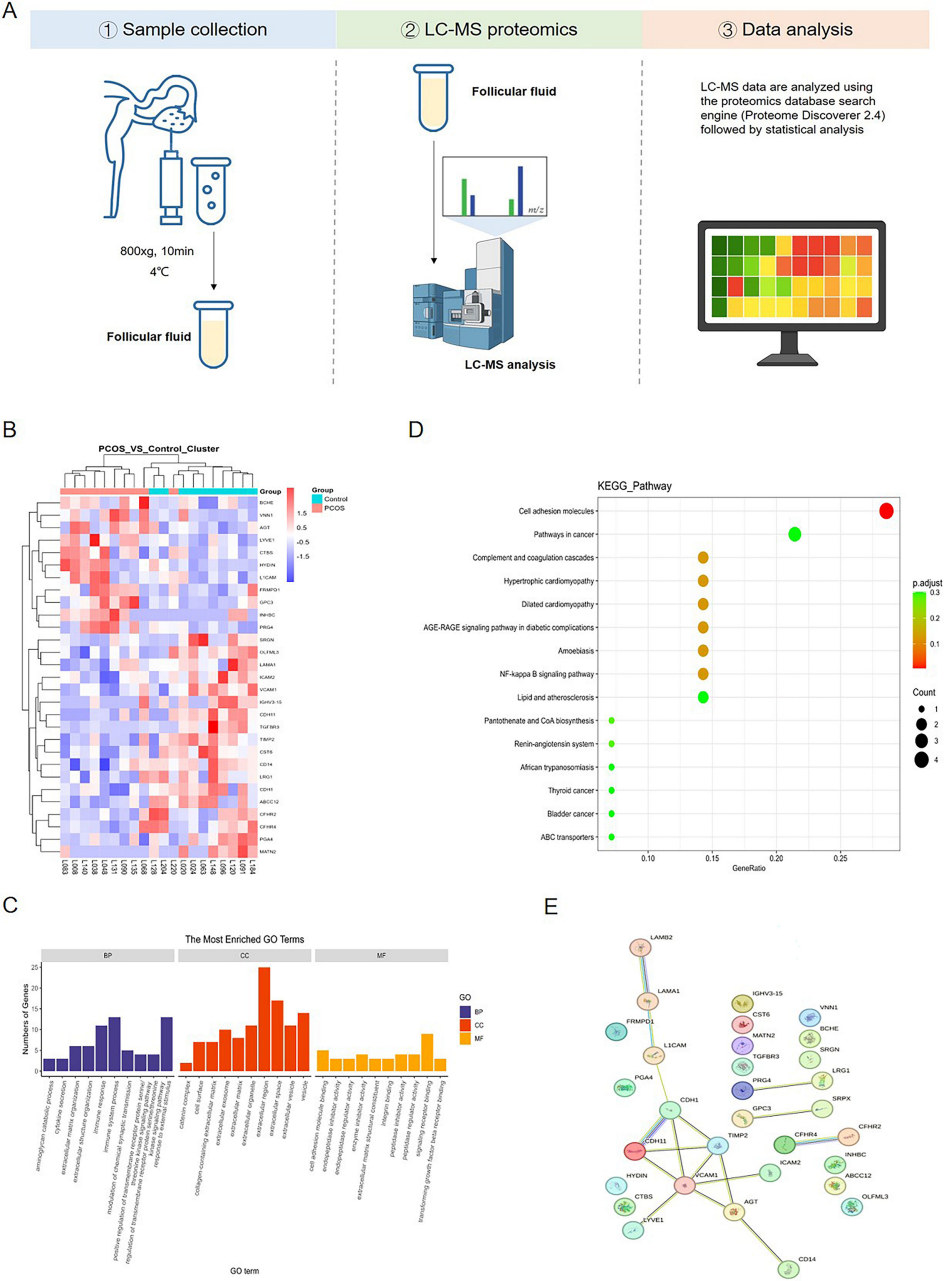
Proteomics profiles between PCOS patients and controls. **(A)** Flow diagram of follicular fluid collection and the corresponding label-free proteomic sequencing workflow. **(B)** Unsupervised hierarchical clustering of all differentially expressed proteins (DEPs) in 20 subjects. ‘Red’ indicates higher expression, and ‘Blue’ indicates lower expression. **(C)** Top 10 Gene Ontology (GO) enrichment terms of DEPs analysis between PCOS and control groups. BP, biological process; CC, cellular component; MF, molecular function. **(D)** Statistical analysis of KEGG pathway enrichment analysis of co-dysregulated proteins. The bubble size represents the number of genes annotated by the DEPs, and the color corresponds to the adjusted *p*-value. **(E)** The protein-protein interaction (PPI) network of DEPs constructed using STRING and visualized with Cytoscape.

To better investigate the potential factors contributing to the reduced oocyte quality in PCOS patients, we conducted a label-free proteomic analysis of follicular fluid from 10 PCOS patients and 10 controls. The flowchart of follicular fluid sample collection and proteomic sequencing analysis is shown in **Figure 1A**. We compared the clinical baseline characteristics of these groups. As shown in **Table 3**, there were no statistically significant differences in age, BMI, FSH, luteinizing hormone (LH), total testosterone (T), and thyroid stimulating hormone (TSH) between total PCOS and total control groups, while AMH levels were higher in women with PCOS compared to controls (7.2 ± 3.84 vs. 4.3 ± 2.21 ng/mL, *p* = 0.050). There was no significant difference between HC and LC subgroups respect to age, BMI, basal FSH, basal LH, total T, TSH, and AMH levels. However, the LH/FSH ratio was significantly higher in the HP group than in the HC group (1.45 ± 0.31 vs. 0.98 ± 0.31, *p* = 0.043), and LH levels were higher in the HP group than in the LP group (9.3 ± 2.63 vs. 5.9 ± 1.38, *p* = 0.042). After normalization and scaling of protein expressions, we found that most PCOS and control samples were clustered into two sub-trees through unsupervised hierarchical clustering. Proteins with an adjusted *P*-value < 0.05 were categorized as differentially expressed. 28 DEPs were identified in this study, with 11 being upregulated and 17 downregulated in the PCOS group compared to the control group. For the clustering analysis, the 28 DEPs were divided into two clusters (**Figure 1B**). The annotations and the quantitative information of these DEPs are shown in **Table 4**. Subcellular prediction was used to characterize the subcellular localization of these DEPs. The GO enrichment analysis demonstrated that most of the upregulated proteins in the biological process classification were involved in regulating the aminoglycan catabolic process, cytokine secretion, extracellular matrix organization, and immune response (**Figure 1C**). Many upregulated proteins were associated with catenin complex, cell surface, collagen-containing extracellular matrix, and extracellular exosome (**Figure 1C**). For cellular components, the DEPs were implicated in cell adhesion molecule binding, endopeptidase inhibitor activity, endopeptidase regulator activity, and enzyme inhibitor activity (**Figure 1C**). KEGG pathway enrichment analysis demonstrated that the DEPs were mainly enriched in pathways related to cell adhesion molecules, pathways in cancer, complement and coagulation cascades, and hypertrophic cardiomyopathy (**Figure 1D**). Furthermore, to identify hub proteins with potential regulatory roles and to explore functional interactions involved in follicular fluid microenvironment homeostasis and oocyte developmental potential in PCOS, we performed a protein-protein interaction (PPI) network analysis using STRING and Cytoscape. This analysis revealed potential interactions among the DEPs. A total of 10 DEPs exhibited direct interactions within the network, including 3 upregulated proteins and 7 downregulated proteins. These interactions are represented by connecting lines in the network diagram (**Figure 1E**). Notably, CDH1, CDH11, and VCAM1 were identified as hub genes within the PPI network, which were closely associated with cell adhesion and intercellular communication (**Figure 1E**).

**Table 3:** Clinical baseline characteristics of patients for proteomics.

Characteristics	HC (n=5)	LC (n=5)	control (n=10)	HP (n=5)	LP (n=5)	PCOS (n=10)	*P*-value				
Maternal age (y)	31.0±3.00	30.0±2.35	30.5±2.59	30.2±2.86	31.4±5.81	30.8±4.37	0.574^a^	0.678^b^	0.690^c^	0.631^d^	0.854^e^
Maternal BMI (kg/m2)	20.3±1.95	20.4±2.47	20.4±2.10	23.4±2.71	21.5±2.40	22.4±2.63	0.935^a^	0.074^b^	0.263^c^	0.518^d^	0.067^e^
Basal FSH level (IU/L)	7.7±2.48	6.9±2.05	7.3±2.19	6.7±2.50	5.8±0.99	6.3±1.86	0.594^a^	0.553^b^	0.479^c^	0.335^d^	0.275^e^
Basal LH level (IU/L)	7.5±3.87	7.0±4.15	7.3±3.79	9.3±2.63	5.9±1.38	7.6±2.68	0.850^a^	0.424^b^	0.042^c^	0.582^d^	0.835^e^
LH/FSH	0.98±0.31	1.0±0.27	0.97±0.28	1.45±0.31	1.04±0.28	1.24±0.36	0.966^a^	0.043^b^	0.058^c^	0.717^d^	0.075^e^
Total T (ng/mL)	0.3±0.09	0.2±0.12	0.3±0.13	0.9±0.91	0.4±0.33	0.7±0.76	0.083^a^	0.397^b^	0.300^c^	0.561^d^	0.165^e^
TSH (μIU/mL)	3.5±1.33	3.1±2.87	3.2±2.15	1.8±1.06	2.4±1.83	2.1±1.37	0.841^a^	0.284^b^	0.686^c^	0.740^d^	0.310^e^
Fasting glucose(mmol/L)	6.0±1.09	6.5±0.89	6.3±0.34	5.4±0.40	5.4±1.40	5.4±0.92	0.950^a^	0.335^b^	0.986^c^	0.575^d^	0.119^e^
Fasting insulin (μU/mL)	11.2±7.37	18.2±9.89	14.7±4.95	16.2±10.33	12.8±7.41	14.7±8.80	0.976^a^	0.679^b^	0.575^c^	0.558^d^	0.998^e^
AMH (ng/mL)	4.5±2.63	4.1±1.90	4.3±2.21	7.8±2.73	6.8±4.97	7.2±3.84	0.783	0.085^b^	0.638^c^	0.343^d^	0.050^e^

BMI, body mass index; FSH, follicle stimulating hormone; LH, luteinizing hormone; T, testosterone; TSH, thyroid stimulating hormone; AMH, anti-Müllerian hormone.

^a^HC vs. LC; ^b^ HC vs. HP; ^c^ HP vs. LP; ^d^ LC vs. LP; ^e^ total PCOS vs. total control.

**Table 4:** Information of differentially expressed proteins in patients with and without PCOS.

Protein accession	Protein name	Fold change	*P*-value
P55103	Inhibin beta C chain	3.3456	0.04499
O95497	Pantetheinase	2.8253	0.04637
Q92954	Proteoglycan 4	1.6632	0.02355
P01019	Angiotensinogen	1.3971	0.0228
Q5SYB0	FERM and PDZ domain-containing protein 1	1.393	0.03417
Q9Y5Y7	Lymphatic vessel endothelial hyaluronic acid receptor 1	1.3507	0.03611
P51654	Glypican-3	1.3309	0.02813
Q4G0P3	Hydrocephalus-inducing protein homolog	1.3296	0.04654
P06276	Cholinesterase	1.3205	0.04134
Q01459	Di-N-acetylchitobiase	1.3197	0.04178
P32004	Neural cell adhesion molecule L1	1.2614	0.04164
P08571	Monocyte differentiation antigen CD14	0.81062	0.00972
P02750	Leucine-rich alpha-2-glycoprotein	0.79946	0.02465
Q9NRN5	Olfactomedin-like protein 3	0.78526	0.04321
P25391	Laminin subunit alpha-1	0.76648	0.04053
P13598	Intercellular adhesion molecule 2	0.74186	0.04736
P19320	Vascular cell adhesion protein 1	0.698	0.03414
P10124	Serglycin	0.58933	0.04859
P12830	Cadherin-1	0.54735	0.01633
P36980	Complement factor H-related protein 2	0.52939	0.02747
Q92496	Complement factor H-related protein 4	0.52836	0.04538
P16035	Metalloproteinase inhibitor 2	0.5282	0.03789
Q15828	Cystatin-M	0.51277	0.04214
P0DJD7	Pepsin A-4	0.50579	0.0041
P55287	Cadherin-11	0.42397	0.04487
Q96J65	Multidrug resistance-associated protein 9	0.40779	0.00195
A0A0B4J1V0	Immunoglobulin heavy variable 3-15	0.29568	0.047
Q03167	Transforming growth factor beta receptor type 3	0.23359	0.01063

### 3.3 Functional enrichment analysis of DEPs associated with oocyte quality

**Figure 2:**
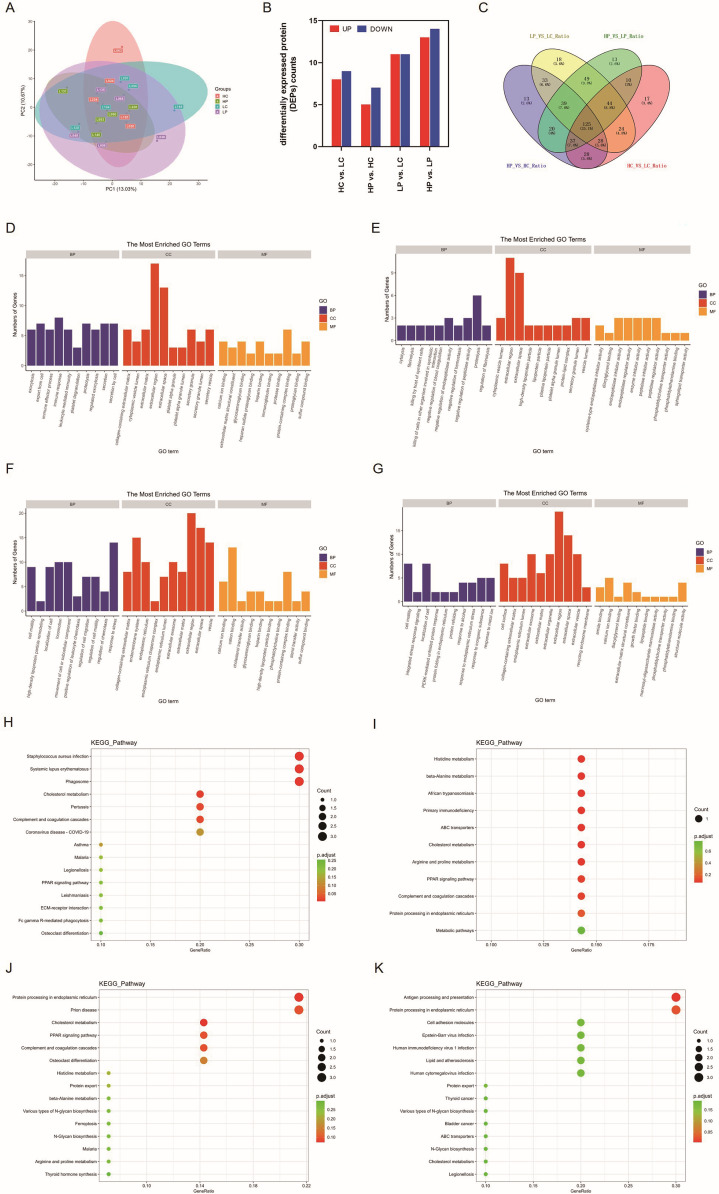
Functional enrichment analysis of DEPs associated with oocyte quality. **(A)** PCA plot of label-free proteomics in the follicular fluid from controls with a high rate of high-quality D3 embryos (HC), controls with a low rate of high-quality D3 embryos (LC), PCOS with a high rate of high-quality D3 embryos (HP), and PCOS with a low rate of high-quality D3 embryos (LP). **(B)** A bar chart showing the numbers of DEPs in pairwise comparisons between the groups (HC vs. LC, HP vs. HC, LP vs. LC, HP vs. LP). **(C)** A Venn diagram showing the number of DEPs in each comparison, and the overlaps between the four main comparison groups. **(D-G)** Bar charts representing the top 10 terms of protein enrichment analysis in HC versus LC **(D)**, HC versus HP **(E)**, HP versus LP **(F)**, and LC versus LP **(G)**. **(H-K)** Bubble plots illustrating the statistical analysis of KEGG pathway enrichment in HC versus LC **(H)**, HC versus HP **(I)**, HP versus LP **(J)**, and LC versus LP **(K)**. The bubble size represents the count of genes annotated by the DEPs, and the color corresponds to the adjusted *p*-value.

The term “oocyte quality” refers to the developmental potential of the oocyte, encompassing multiple morphological markers of MII oocytes, such as zona pellucida, spindle apparatus, and vacuolization. Despite this, the mechanisms of oocyte cytoplasmic maturation are still poorly understood. To investigate the potential cytoplasmic markers of oocyte maturation, we conducted a more in-depth analysis of the proteomic sequencing results categorized according to the quality rate of D3 embryos. Principal component analysis (PCA) revealed markedly differences between HP, LP, LC and HC clusters (**Figure 2A**). DEPs in the FF were identified through pairwise comparisons, with 12, 22, 27, and 17 DEPs were identified between HC and LC, HP and HC, LP and LC, HP and LP, respectively (**Figure 2B**). The Venn diagram describes the intersections and unions of DEPs in each comparison group (**Figure 2C**), with the numbers in the overlapping parts of the circles representing the number of DEPs common to each comparison.

GO and KEGG pathway enrichment analyses were applied to identify key proteins and pathways contributing most to the different patterns associated with oocyte quality in patients with or without PCOS. GO enrichment analysis of biological processes, cellular components and molecular function was performed to evaluate the functional significance of these candidate proteins. Exocytosis, export from cell, and immune effector process were the top three biological processes between HC and LC groups (**Figure 2D**). In terms of cellular components, proteins showed high enrichment in the collagen-containing extracellular matrix between HC and LC groups (**Figure 2D**), whereas in cytoplasmic vesicle lumen between HP and HC groups (**Figure 2E**). Interestingly, the DEPs exhibited a similar pattern with a high enrichment in high-density lipoprotein particles in both HP vs. LP and HP vs. HC comparisons (**Figures 2E, F**), while a high enrichment in cell motility and cell surface was observed between LP and LC (**Figure 2G**). Furthermore, it is worth noting that DEPs were enriched in cholesterol metabolism in all comparison groups through KEGG pathway analysis (**Figures 2H–J**), which suggested that cholesterol metabolism may play an important role in the microenvironment of oocyte maturation and development. It is worth mentioning that in the LP and LC groups, only the antigen processing and presentation and protein processing in endoplasmic reticulum pathways showed significant differences when using adjusted p-values for KEGG enrichment analysis (**Figure 2K**).

### 3.4 Construction of weighted gene co-expression networks and identification of key modules

**Figure 3:**
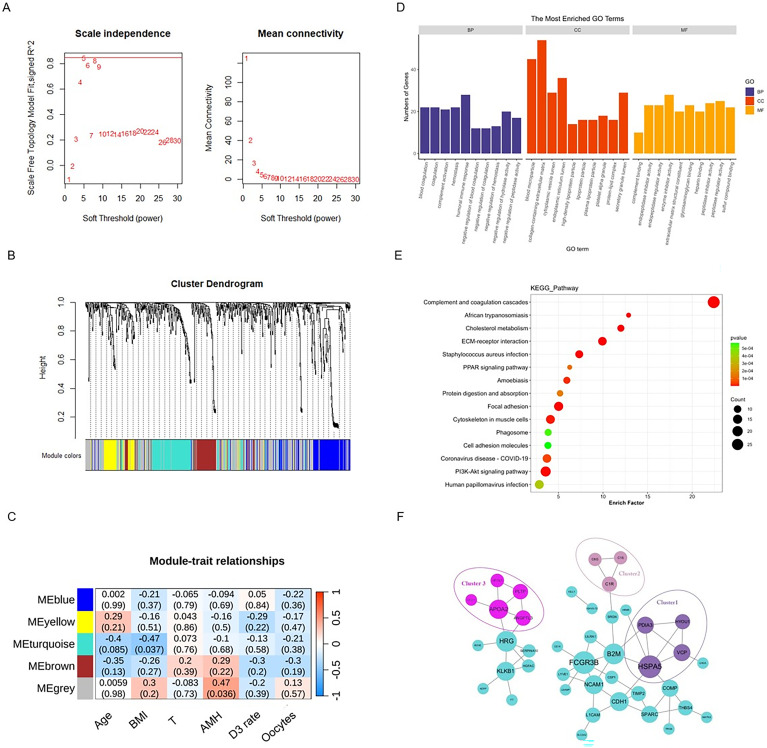
Weighted co-expression network construction and identification of key modules. The proteins identified in the proteomic analysis were further analyzed using the R package WGCNA. First, the function of the R package “WGCNA” is used to check whether the proteins of the samples need to be filtered and to select a suitable soft threshold. Then, the co-expression network was constructed by setting the minimum number of proteins per module to 300 according to the criteria of the hybrid dynamic tree-cutting algorithm. Finally, Pearson correlation coefficients were used to analyze the association of module signature proteins. **(A)** Determination of soft threshold power. **(B)** Dendrogram of all DEPs clustered based on the measurement of dissimilarity. The color band shows the results obtained from the automatic single block analysis. **(C)** Heatmap of the correlation between the module eigengenes and clinical or biochemical characteristics of samples between PCOS and controls. **(D)** Bar charts representing the top 10 terms of protein enrichment analysis in the DEPs of the gray modules. **(E)** Bubble plots illustrating the statistical analysis of KEGG pathway enrichment in the DEPs of the gray modules. **(F)** The co-expression network of DEPs.

WGCNA was performed to construct co-expressed networks and identify co-expression modules. After selecting the determination of soft-thresholding power (R2 = 0.8), hierarchical clustering of the samples was performed based on a Euclidean distance computed on log_10_-transformed proteomic sequencing fractional counts. We profiled 612 proteins from these 20 samples using WGCNA, and 5 protein co-expression modules were identified (**Figures 3A, B**). The modular dynamic protein expression pattern showed that proteins in the grey module were strongly correlated with AMH, high-quality embryo rate, and the number of oocytes retrieved (**Figure 3C**). To further clarify the biological functions of hub proteins, GO functional and KEGG pathway enrichment of hub proteins in the gray module was analyzed. Interestingly, the DEPs in the gray module were significantly enriched in endoplasmic reticulum stress and cholesterol metabolism (**Figures 3D, E**), suggesting that the decreased quality of oocytes and embryos in PCOS patients may be related to endoplasmic reticulum stress and cholesterol metabolism disorders in the follicular fluid microenvironment. Furthermore, we conducted a co-expression network analysis of the DEPs and identified three clusters. The functions of the proteins in these three clusters were mainly focused on endoplasmic reticulum stress, fatty acid metabolism, and cholesterol metabolism (**Figure 3F**). The proteins within these three clusters encompass APOA2 and PLTP (associated with lipid and cholesterol metabolism), C1R (an initiator of the classical complement pathway), and HSPA5 and HYOU1 (related to endoplasmic reticulum stress) (**Figure 3F**). Among them, the expression levels of PLTP and HYOU1 show significant differences between the PCOS and control groups. Overall, these results suggested that abnormal cholesterol metabolism and excessive endoplasmic reticulum stress in the follicular fluid environment may have an adverse effect on the quality of oocytes in PCOS.

### 3.5 Validation of hub protein expression associated with oocyte quality

**Figure 4:**
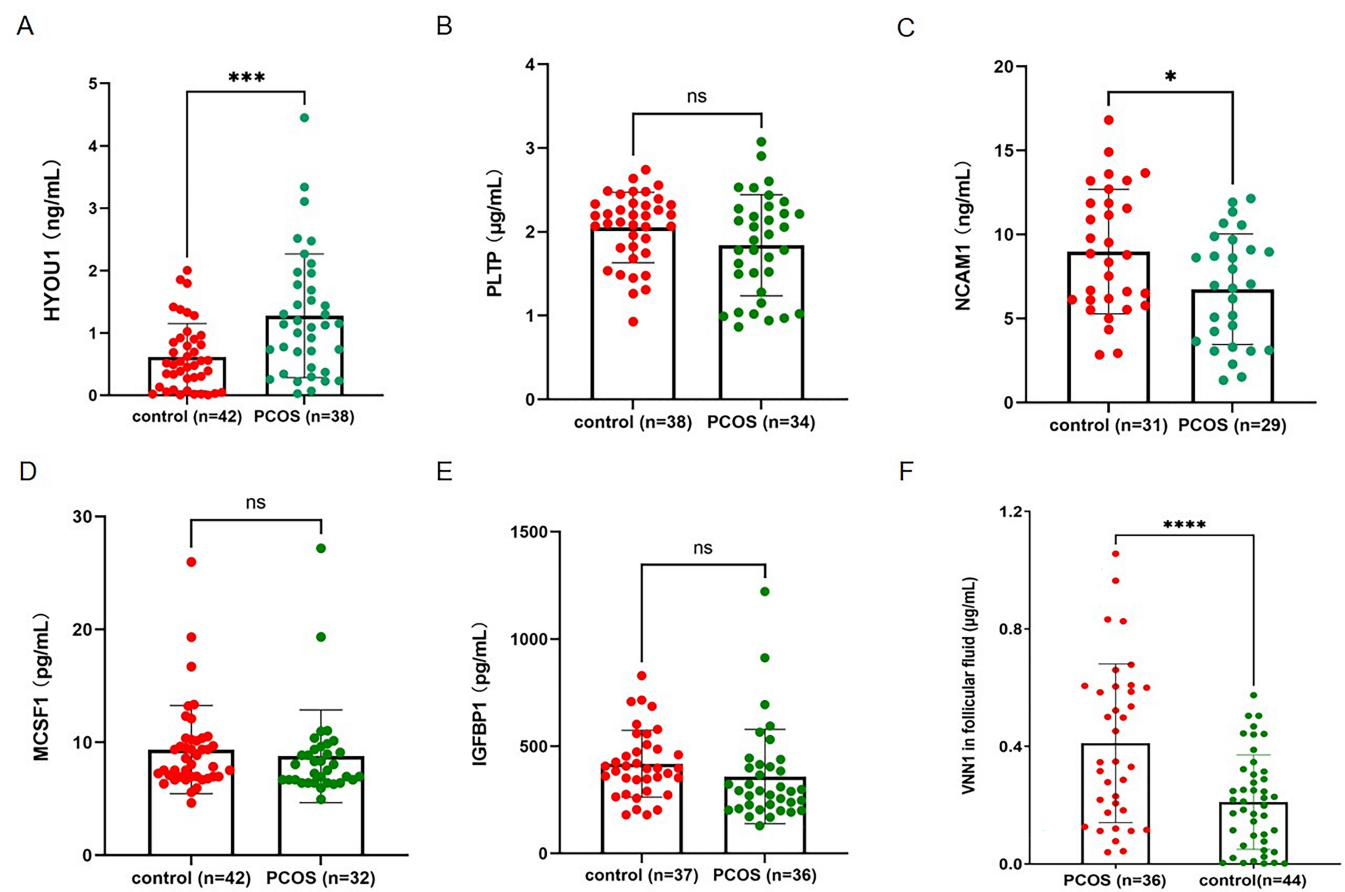
Validation of hub protein concentration levels in follicular fluid associated with oocyte quality by ELISA. **(A-F)** The differential concentration levels of HYOU1 **(A)**, PLTP **(B)**, NCAM1 **(C)**, MCSF1 **(D)**, IGFBP1 **(E)**, and VNN1 **(F)** were further validated using follicular fluid samples collected from 38 controls and 42 PCOS patients. The concentrations of selected proteins in follicular fluid samples were measured by commercial ELISA kit. Statistical significance is denoted by *p < 0.05, ***p < 0.001, ****p < 0.0001.

ELISA was used to validate the differential abundance of DEPs in follicular fluid. Based on the results of bioinformatic analysis and WGCNA results, 6 representative DEPs were selected for ELISA validation in the FF samples from 42 PCOS patients and 38 controls to comprehensively investigate the potential mechanism of oocyte maturation. The selected DEPs include HYOU1, PLTP, VNN1, macrophage colony-stimulating factor 1 (MCSF1), neural cell adhesion molecule 1 (NCAM1), and insulin-like growth factor binding protein 1 (IGFBP1). We selected these DEPs for validation mainly based on the P - values in proteomic data and their functions in key biological pathways relevant to our research. Additionally, proteins with well - characterized functions and available reliable ELISA antibodies were also included. The selected DEPs included proteins involved in endoplasmic reticulum stress (HYOU1), cholesterol metabolism (PLTP), cell adhesion (NCAM1), cytokine interaction (MCSF1), oxidative stress (VNN1) and regulation of cell communication (IGFBP1). The ELISA assay results indicated a significant decrease in NCAM1 protein levels and a significant increase in VNN1 and HYOU1 in PCOS patients compared with controls, while IGFBP1, MCSF1 and PLTP protein levels did not differ significantly between the two groups (**Figures 4A–F**).

### 3.6 Association between oocyte quality and abnormal PLTP and cholesterol metabolism in follicular fluid of PCOS patients

**Figure 5:**
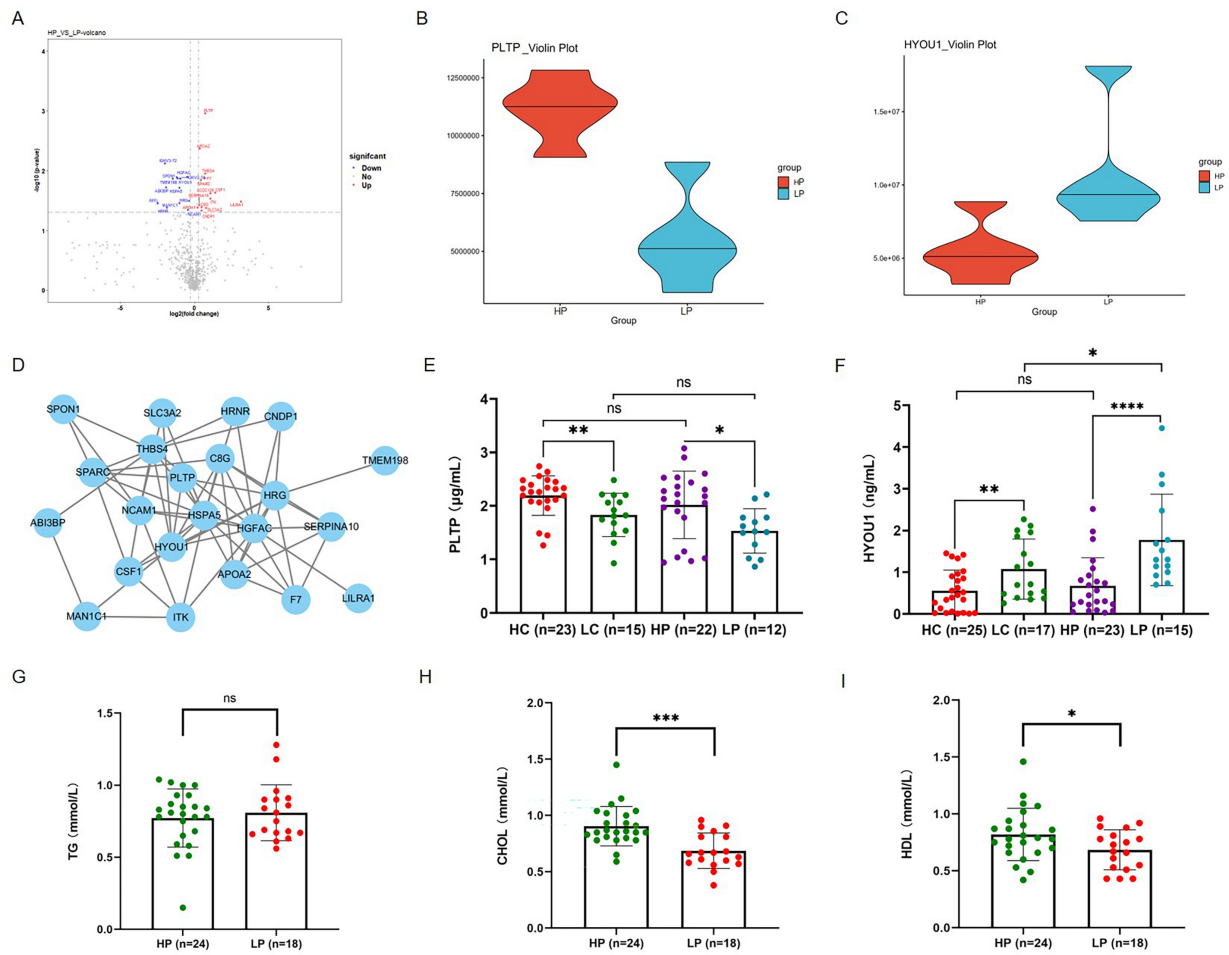
Oocyte quality declines in association with abnormal PLTP and cholesterol metabolism of follicular fluid in PCOS patients. **(A)** Volcano plot of DEPs between HP and LP group. Up- and downregulated DEPs with *p* < 0.01 and fold change > 2 are highlighted in light red and light blue, respectively. **(B)** Proteomic sequencing results for PLTP expression in five PCOS patients with high rate of high-quality D3 embryos (HP) group and five PCOS patients with low rate of high-quality D3 embryos (LP) group. **(C)** Proteomic sequencing results for HYOU1 expression in HP group and LP group. **(D)** The co-expression network of DEPs in HP group and LP group. **(E)** ELISA assay analysis of PLTP protein levels in the follicular fluid between HP and LP groups. **(F)** ELISA assay analysis of HYOU1 protein levels in the follicular fluid between HP and LP groups. **(G-I)** The expression levels of triglycerides (TG) **(G)**, total cholesterol (CHOL) **(H)**, and high-density lipoprotein (HDL) **(I)** in follicular fluid between HP and LP groups. Statistical significance is denoted by *p < 0.05, **p < 0.01, ***p < 0.001.

To investigate the correlation between abnormal cholesterol metabolism and oocyte quality, we analyzed the expression levels of DEPs in the FF between PCOS patients with high rate of high-quality D3 embryos (HP) and those with low rate of high-quality D3 embryos (LP) (**Figure 5A**). Our proteomic analysis revealed that the expression of PLTP was significantly upregulated in the follicular fluid of the HP group, whereas the expression of HYOU1 was significantly downregulated (**Figures 5B, C**). Furthermore, we performed a PPI analysis using STRING and Cytoscape to elucidate potential proteins among the DEPs and related intact proteins between the HP and LP group (**Figure 5D**). ELISA assay analysis confirmed significantly higher expression levels of PLTP protein in the HP group compared with the LP group (**Figure 5E**). However, the expression of HYOU1 was significantly downregulated in the HP group (**Figure 5F**). Interestingly, the expression trends of PLTP and HYOU1 were consistent in both the PCOS group and the control group, with higher expression of PLTP and lower expression of HYOU1 observed in the HC group (**Figures 5E, F**). To further determine whether these differences are related to oocyte quality, we analyzed the expression levels of triglycerides (TG), total cholesterol (CHOL), and high-density lipoprotein (HDL) in follicular fluid between HP and LP groups. The results showed that the CHOL and HDL levels were significantly upregulated in follicular fluid of PCOS patients with high rate of high-quality D3 embryos compared with those with low rate of high-quality D3 embryos, while the TG levels showed no significant difference between the two groups (**Figures 5G–I**). These results suggest that cholesterol metabolism may play an important regulatory role in the microenvironment of follicular fluid, leading to the poor quality of oocytes in PCOS patients.

## 4 Discussion

PCOS is a complicated endocrine disorder characterized by metabolic, reproductive, and psychological problems. PCOS-related infertility is primarily attributed to impaired ovulation disorders or anovulation. Nevertheless, despite the medical induction of ovulation and the retrieval of a greater number of oocytes, PCOS patients persistently exhibit a reduced rate of high-quality embryo formation. The determinants influencing oocyte quality in PCOS have yet to be completely understood, and the identification of higher quality oocytes continues to pose a significant challenge within the assisted reproductive technology. In this study, a label-free quantitative proteomic analysis was employed to investigate the proteomic profiles in the follicular fluid of women with PCOS. We identified 28 DEPs, with 11 upregulated and 17 downregulated in the PCOS group compared to the control group. GO terms and significant pathways associated with the DEPs were also identified, including aminoglycan catabolic process, cytokine secretion, extracellular matrix organization, and immune response, which were dysregulated in the FF of women with PCOS. KEGG pathway enrichment analysis demonstrated that the DEPs were mainly enriched in cell adhesion molecules, pathways in cancer, complement and coagulation cascades, and hypertrophic cardiomyopathy. Furthermore, we conducted an analysis of the differentially expressed proteins in follicular fluid, categorized according to the rates of high-quality D3 embryos. The findings indicated that exocytosis, cholesterol metabolism, and immune effector process were the differential biological processes associated with oocyte quality.

A follicle is composed of a germ cell and its surrounding granulosa cells and theca cells. Follicular development begins with the primordial follicle, which is formed by a primary oocyte and a single surrounding layer of flattened granulosa cells during fetal life and later develops into primary, secondary and antral follicles (21). Meanwhile, the follicular development involves a complex process of oocyte growth, the proliferation of granulosa and theca cells and the formation of FF. The follicular fluid that fills the antrum of growing ovarian follicles provides an important microenvironment for oocyte development (22, 23). FF contains metabolites, proteins, hormones, and other components that support oocyte growth, maturation, and acquisition of developmental competence (24). There is an intimate metabolic cooperation between the oocyte and surrounding granulosa cells, facilitated by the exchange of nutrients and signaling molecules via the FF (25). Disturbances in FF composition may adversely affect oocyte quality and preimplantation embryo development, contributing to poorer IVF outcomes in women with PCOS. Our results showed that many of the upregulated proteins in PCOS patients were associated with catenin complex, extracellular exosome, and collagen-containing extracellular matrix. The catenin signaling transduction pathway, participating in regulating proliferation and cell fate determination, is essential for early embryonic development and the appropriate activation of the female reproductive system, as well as the regulation of hormonal activity in ovarian granulosa cells (26). Previous study has demonstrated the dysregulated expression profile of WNT/β-catenin signaling genes in human oocytes obtained from PCOS patients (27). Communication between somatic cells and the oocyte is essential for the appropriate development of follicles and the maturation of oocytes, and disruptions in this finely tuned microenvironment can result in ovarian disorders, including PCOS (28). Recent studies have confirmed that the intercellular communication between somatic cells and oocytes is partially mediated by extracellular exosomes, which are spherical entities characterized by a lipid bilayer structure (29–31). Our investigation identified MCSF1 and IGFBP1 as the anomalous protein associated with exosomes in the FF of patients with PCOS. CSF1 has previously been confirmed to be a target gene for the action of microRNA in ovarian exosomes (32), which may regulate the function of ovarian granulosa cells and macrophages (33). Recent researches have demonstrated levels of IGF1 in FF were associated with IVF pregnancy outcome (34) and microRNAs from exosomes disrupted the expression of IGFBP1 (35). However, our ELISA validation analysis revealed that the expression levels of MCSF1 and IGFBP1 in the FF of patients with PCOS did not exhibit statistically significant differences compared to the control group following by the ELISA validation. It is still unclear whether MCSF1 and IGFBP1 in the FF are secreted by exosomes and what their specific roles may be in follicular development.

PCOS is distinguished by persistent low-grade inflammation and is linked to various autoimmune disorders (36). In our study, some dysregulated proteins in PCOS patients were enriched in the aminoglycan catabolic process, cytokine secretion, extracellular matrix organization, and immune response. Previous research has demonstrated elevated levels of amino acids such as leucine, isoleucine, and valine in the FF of PCOS patients (37). Another study using NMR metabolomics revealed decreased levels of alanine and glutamine in the FF of PCOS patients. Further analysis indicated a significant positive correlation between glutamine levels and top-quality embryo rate, suggesting that higher glutamine levels in FF may enhance embryo quality in PCOS patients (38). The imbalance between proinflammatory and anti-inflammatory factors creates an inflammatory microenvironment within the follicle that could adversely affect oocyte maturation and developmental competence (39). Previous studies have reported that levels of tumor necrosis factor alpha (TNFα) and interleukin 6 (IL-6) were significantly higher in the FF of PCOS patients compared to controls, while IL-23 levels were significantly lower in PCOS patients (40). These findings suggest that the disturbed follicular microenvironment in PCOS patients may be detrimental to oocyte maturation and developmental competence. Our findings additionally indicated that the expression levels of NCAM1 in the follicular fluid of PCOS patients were significantly decreased. NCAM1 (CD56), which is implicated in intercellular and cell-matrix interactions during developmental and differentiation processes, contributes significantly to the proliferation of T lymphocytes, B lymphocytes, and natural killer (NK) cells (41), as well as in the extracellular matrix (ECM) of the cumulus oocyte complex (COC) during follicle development (42). Previous studies have demonstrated that the lower levels of CD56^+^NKG2D^+^NK cells were associated with a poor postoperative pregnancy rate and a delayed time to successful pregnancy in ovarian endometrioma patients (43) and the downregulation of NCAM1 in cumulus cells seems to be related to PCOS (44). Our previous study also found that the dysregulation of CD56^+^NK cells in the endometrium of PCOS patients (45). However, further investigation is required to elucidate the specific role of NCAM1 in oocyte development among individuals with PCOS.

High-quality oocytes have a high level of intrinsic ability to undergo meiotic division and achieve successful pregnancy (24). As a noninvasive examination, analyzing the FF provides a window into the intrafollicular milieu and identifying abnormalities in its composition may reveal pathways disturbed in PCOS that lead to poorer oocyte quality and IVF outcomes (37). To date, only a small proportion of the proteome from the FF of patients with PCOS has been revealed. One study compared the proteomic profiles of FF from several hyper-stimulated women at oocyte retrieval with those of three women before ovulation trigger, two of whom were hyper-stimulated and one with no apparent stimulation (46). Another study has utilized iTRAQ-based analysis to compare the protein profiles of FF from Indian PCOS patients and controls, suggesting that proteins involved in extracellular matrix remodeling, the complement coagulation cascade, fibrinolysis, vasculature development, angiogenesis, lipid transport, and metabolism were dysregulated in PCOS (15). A TMT-based proteomic analysis identified 41 DEPs between overweight or obese PCOS patients and non-PCOS women, and 19 DEPs between normal-weight PCOS patients and non-PCOS women (13). Despite the advancements in identifying of novel proteins and pathways potentially involved in the pathogenesis of PCOS in recent years, it remains challenging to pinpoint specific proteins that contribute to the poor oocyte quality of PCOS. This research represents the inaugural investigation into the screening of follicular fluid markers that are directly associated with oocyte quality in PCOS patients, categorized according to the rates of high-quality D3 embryos. Our findings suggested that abnormal cholesterol metabolism, oxidative stress, and endoplasmic reticulum stress may play significant roles in the deterioration of oocyte quality in PCOS.

Cholesterol, which is abundantly found in numerous tissues, serves as a crucial component for maintaining tissue structure, supporting vital biological functions, and facilitating metabolic processes. The implications of cholesterol in female reproduction have been suggested by various studies across different species (47–51). Studies across different species have highlighted the significance of cholesterol in female reproduction (48–51). PCOS is characterized by dysregulated adipogenesis and lipolysis, both of which are essential processes for the proper maturation of oocytes and the development of embryos (52). These impairments may lead to enhanced aromatization of androgens to estrogens, and altered gonadotropin secretion (53, 54). Cholesterol, the precursor for steroid hormones productions (55), is essential for the development and maturation of follicles. Ovarian cholesterol is mainly derived from blood lipoproteins and *de novo* synthesis by follicular cells (56). Despite the importance of cholesterol metabolism in follicular development, recent studies investigating its effects are limited. The present study revealed that cholesterol metabolism is markedly enriched in all comparison groups, indicating its crucial role in the microenvironment associated with follicular development. Our results demonstrated a significant difference in the follicular fluid cholesterol levels between the HP and LP, which failed to be corroborated between HC and LC. Furthermore, through bioinformatics analysis and subsequent validation via ELISA, we identified the pivotal differential protein, PLTP, which modulates cholesterol metabolism within follicular fluid. We also conducted an analysis to explore the relationship between the expression levels of PLTP and the oocyte quality. PLTP was first described as a plasma factor facilitating the transfer of phospholipid between lipoproteins and plays multiple roles in lipoprotein metabolism (57). The levels of PLTP mRNA remain consistent across different stages of estrus cycles, suggesting that steroid hormones do not significantly influence gene expression (58). PLTP facilitates the transfer of amphiphilic lipids among circulating lipoproteins as well as between lipoproteins, cells, and tissues and plays a crucial role in regulating the plasma concentrations, turnover rates, and functional properties of the major classes of lipoproteins, which include very low-density lipoproteins (VLDL), low-density lipoproteins (LDL), and HDL (59, 60). Evidence suggests that FF HDL mainly originates from plasma, either directly or through remodeling in the follicular antrum (61). In this study, we found that the FF from patients with PCOS exhibited elevated TC levels and reduced HDL levels. Previous studies demonstrated that FF cholesterol concentrations are positively correlated with follicular size, and the proportions of LDL and VLDL in preovulatory follicles increase gradually (62). Unfortunately, we failed to measure the LDL levels in the sample solution as a result of their extremely low concentrations. Moreover, cholesterol was proposed to engage the inflammasome pathway in local macrophages (63). Our previous study found that M1 macrophages were significantly upregulated in the FF of PCOS patients (64), which further suggested that the altered cholesterol metabolism within the FF of PCOS may contribute to irregularities in the microenvironment associated with follicular development.

Oxidative stress is characterized by an imbalance within the redox system, wherein the concentration of free radicals rises to a degree that surpasses the neutralizing capacity of the endogenous antioxidant mechanisms (65). Oxidative stress may serve as a primary factor in oocyte aging and reproductive pathologies (66), which is particularly associated with abnormal follicular atresia, irregularities in meiosis, reduced fertilization rates, delayed embryonic development, and various reproductive disorders, such as PCOS and endometriosis-related ovarian cysts (67). VNN1 is anchored to the cellular membrane with the pantetheine hydrolase activity, which hydrolyzes pantetheine to produce cysteamine and promote oxidative stress and the inflammatory response (68). Our findings indicate that the expression levels of VNN1 in the follicular fluid of PCOS patients were markedly increased, implying the presence of oxidative stress within the microenvironment of follicular development in this patient population.

Endoplasmic reticulum (ER) stress, characterized by the accumulation of unfolded or misfolded proteins within the ER, arises from a range of physiological and pathological conditions that either elevate the demand for protein folding or diminish the protein-folding capacity of the ER (69). Prior research has indicated that ER stress is significantly activated in the granulosa cells of follicles during the later stages of development (70), with a more pronounced effect observed in PCOS patients and in murine models, compared to non-PCOS control subjects (71). Excess testosterone induces ER stress in granulosa cells of PCOS patients (72), and administration of an ER stress inhibitor to PCOS-afflicted mice resulted in a reduction of apoptosis in granulosa cells (73). Most proteins are synthesized and processed in the ER before becoming functional (74). In this study, we observed a significant decrease of HYOU1 protein levels in PCOS patients compared with controls. HYOU1 encodes a chaperone protein situated within the ER. The expression of HYOU1 is notably elevated in a range of pathological conditions, including various forms of cancer and diseases associated with ER stress (75). HYOU1 is recognized not only for its significant protective function in the initiation and progression of tumors but also as a promising therapeutic target in oncology (76). Furthermore, due to its capacity to elicit an anti-tumor immune response, HYOU1 may serve as an immune-stimulatory adjuvant and a molecular target for the treatment of numerous endoplasmic reticulum-related disorders (77, 78). Currently, there is a lack of fundamental research regarding HYOU1 and ER stress in reproductive studies. Our findings offer valuable insights for investigating oocyte development and the establishment of the follicular microenvironment. Further exploration of the effects of ER stress on oocyte quality could be crucial for improving outcomes in assisted reproductive technologies.

Strengths of this study were the well-matched groups of women with and without PCOS, who were all undergoing *in vitro* fertilization and were subjected to an identical antagonist ovulation induction protocol, which may significantly minimize the variations in the microenvironment and composition of follicular fluid that may arise from differing ovulation induction protocols. The fact that the study was based upon follicular fluid samples as opposed to peripheral blood samples is also a major strength. An additional advantage is that our follicular fluid assessments are derived from pooled follicles, allowing for a strong correlation with the pregnancy outcomes of the respective population. Limitations were that this was a relatively small study and the women with PCOS did not have the consistent PCOS phenotype in terms of oligo-ovulation or anovulation, clinical and/or biochemical hyperandrogenism, and polycystic ovarian morphology as observed by ultrasound, and this may have reduced differences in their protein profiles when compared with the control women. Furthermore, the control group included, patients with tube factor infertility, which refer to infertility caused by abnormalities of fallopian tubes, such as tubal obstruction or damage. Although these patients served as controls in this study, underlying reproductive conditions may still influence certain physiological processes and should be considered when interpreting the results. Moreover, in addition to the intrinsic quality of the oocyte and the microenvironment of the follicular fluid, sperm-related factors are also pivotal in fertilization and early embryonic development. In this study, follicular fluid samples were obtained from patients undergoing IVF rather than ICSI treatment, in which fertilization and embryo quality are primarily influenced by the characteristics of the oocytes. However, sperm parameters, including motility, morphology, DNA integrity, and fertilization capacity, may independently influence fertilization success as well as the subsequent developmental potential of the embryo. Therefore, a comprehensive assessment that integrates both oocyte quality and sperm function may offer a more thorough understanding of the determinants affecting fertilization outcomes in assisted reproductive technologies, particularly among patients with PCOS.

The visual evaluation of oocyte morphology is the predominant technique employed for the selection of high-quality oocytes. Nevertheless, this method possesses intrinsic limitations, as it does not yield a thorough understanding of the genetic and molecular attributes of the oocytes (79). PCOS patients tend to have more immature oocytes, lower-quality oocytes and embryos, and reduced fertilization rates compared with women without PCOS (80, 81). However, the present evaluation of oocyte quality in PCOS patients remain confined to broad morphological scoring criteria. Furthermore, there is a lack of documented evidence regarding biomarkers associated with oocyte quality within the microenvironment of follicular development. The current study has investigated the potential follicular biomarkers to aid in the selection of high-quality oocytes in PCOS patients. Concurrently, we identified multiple potential biomarkers including PLTP, VNN1 and HYOU1 that may serve as indicators for evaluating oocyte quality.

## Data Availability

The data presented in the study are depositedin the Dryad (http://datadryad.org/) repository (Dataset DOI: 10.5061/dryad.j3tx95xwb).
